# Achieving the Sustainable Development Goals in the post-pandemic era

**DOI:** 10.1057/s41599-022-01283-5

**Published:** 2022-08-06

**Authors:** Wenwu Zhao, Caichun Yin, Ting Hua, Michael E. Meadows, Yan Li, Yanxu Liu, Francesco Cherubini, Paulo Pereira, Bojie Fu

**Affiliations:** 1 State Key Laboratory of Earth Surface Processes and Resource Ecology, Faculty of Geographical Science, Beijing Normal University, Beijing, China; 2 Institute of Land Surface System and Sustainable Development, Faculty of Geographical Science, Beijing Normal University, Beijing, China; 3 Department of Environmental & Geographical Science, University of Cape Town, Rondebosch, South Africa; 4 School of Geographic and Ocean Sciences, Nanjing University, Nanjing, China; 5 College of Environmental Sciences, Zhejiang Normal University, Jinhua, China; 6 Industrial Ecology Program, Department of Energy and Process Engineering, Norwegian University of Science and Technology, Trondheim, Norway; 7 Environmental Management Center, Mykolas Romeris University, Vilnius, Lithuania; 8 State Key Laboratory of Urban and Regional Ecology, Research Center for Eco-Environmental Science, Chinese Academy of Sciences, Beijing, China

**Keywords:** Geography, Development studies

## Abstract

The COVID-19 pandemic continues to pose substantial challenges to achieving the Sustainable Development Goals (SDGs). Exploring systematic SDG strategies is urgently needed to aid recovery from the pandemic and reinvigorate global SDG actions. Based on available data and comprehensive analysis of the literature, this paper highlights ongoing challenges facing the SDGs, identifies the effects of COVID-19 on SDG progress, and proposes a systematic framework for promoting the achievement of SDGs in the post-pandemic era. Progress towards attaining the SDGs was already lagging behind even before the onset of the COVID-19 pandemic. Inequitable distribution of food–energy–water resources and environmental crises clearly threaten SDG implementation. Evidently, there are gaps between the vision for SDG realization and actual capacity that constrain national efforts. The turbulent geopolitical environment, spatial inequities, and trade-offs limit the effectiveness of SDG implementation. The global public health crisis and socio-economic downturn under COVID-19 have further impeded progress toward attaining the SDGs. Not only has the pandemic delayed SDG advancement in general, but it has also amplified spatial imbalances in achieving progress, undermined connectivity, and accentuated anti-globalization sentiment under lockdowns and geopolitical conflicts. Nevertheless, positive developments in technology and improvement in environmental conditions have also occurred. In reflecting on the overall situation globally, it is recommended that post-pandemic SDG actions adopt a “Classification–Coordination–Collaboration” framework. Classification facilitates both identification of the current development status and the urgency of SDG achievement aligned with national conditions. Coordination promotes domestic/international and inter-departmental synergy for short-term recovery as well as long-term development. Cooperation is key to strengthening economic exchanges, promoting technological innovation, and building a global culture of sustainable development that is essential if the endeavor of achieving the SDGs is to be successful. Systematic actions are urgently needed to get the SDG process back on track.

## Introduction

In 2015, the United Nations (UN) adopted “The 2030 Agenda for Sustainable Development” and proposed 17 Sustainable Development Goals (SDGs) with the aim of eradicating poverty and promoting peace and prosperity for all on a healthy planet by 2030 (UN, 2015). While time is running out to achieve the 2030 Agenda, the world is struggling to combat the COVID-19 pandemic. By May 2022, more than 500 million COVID-19 cases were confirmed, with more than six million deaths (WHO, 2022). Health services were stretched to the verge of collapse, lockdowns resulted in labor forces being laid off, and millions pushed back to extreme poverty and malnutrition (Sachs et al., 2020; Stephens et al., 2020).

Even prior to the pandemic, SDG advancement was constrained and delayed, but the health crisis and socio-economic recession resulting from COVID-19 have severely impeded SDG progression. Systematic and practical solutions are urgently needed to get the SDGs back on track. Accordingly, the objectives of this paper are to (1) analyze challenges to SDGs progress that prevailed prior to the outbreak of the COVID-19 pandemic; (2) identify the impact of COVID-19 on progress towards achieving the SDGs; and (3) provide a systematic and practical framework for implementing SDGs in the post-pandemic era.

## Challenges facing SDG progress before COVID-19

### Inequitable access to resources limits sustainable development

Food, energy, and water (SDG 2, 6, 7) are the resource pillars on which the SDG implementation stands (Stephan et al., 2018). Their security is threatened by human development pressures and ecosystem degradation. Since 1970, due to the doubling of the global population (World Bank, 2020), primary energy and food production have more than tripled (IPBES, 2019), yet billions of people still suffer from food and energy shortages, and inadequate water supply in terms of both quantity and quality (Bazilian et al., 2011). With the global population estimated to rise to nine billion by 2050, energy and food production must increase by 50% and 70%, respectively, to meet human basic needs (EIA, 2021; UNEP, 2021). Moreover, one-sixth of human-dominated land has experienced ecosystem service degradation (UNEP, 2021), endangering the supply of food, energy, and water, and therefore undermining the very foundations of sustainable development (Yin et al., 2021).

### Environmental crises threaten SDG implementation

Climate change, ecosystem degradation, and pollution (SDG 13–15) are major global environmental risks that weaken the natural foundations supporting the SDGs (UNEP, 2021). These risks inhibit efforts toward poverty reduction, food and agricultural security, human health, and water security (SDG 1–3, 6). Unstable climate conditions also compromise the safety of urban infrastructures (SDG 9, 11) (Nerini et al., 2019). Ecosystem degradation is detrimental to gender equality, especially in rural areas, where local livelihoods are primarily dependent on ecosystem services and women are discriminated against in terms of access to land and education (SDG 5) (Naidoo and Fisher, 2020; Yin et al., 2022). Increased levels of environmental degradation and pollution also amplify the development gap and accentuate inequalities between countries. Low-income countries, where development is highly dependent on healthy soil, clean water, and climate-sensitive sectors such as agriculture bear the greatest burden of climate change and pollution (SDG 10) (Lusseau and Mancini, 2019; Yin et al., 2021). Progress in sustainable energy, responsible production and consumption, economic growth, and decent work (SDG 7, 8, 12) are all hampered by environmental pollution and the loss of natural capital (IPBES, 2019). About 3.2 billion people are affected by land degradation and pollution, and climate-related natural disasters cost $155 billion in 2018 (Swiss Re Institute, 2019). More than 2500 conflicts worldwide were attributed to environmental crises that exacerbate migration and competition for natural resources (World Economic Forum, 2020), seriously jeopardizing the development of peaceful and inclusive societies (SDG 16, 17).

### Gaps between SDG visions and actual capabilities discourage national efforts

The effective implementation of SDGs in a country depends on their integration into national socio-economic development plans. While the UN is committed to a policy of ‘leaving no one behind’ in achieving the SDGs, only about half the countries (53%) have completed or are developing, a roadmap for SDGs to guide their implementation (Allen et al., 2018). This is largely due to critical gaps between the SDGs’ requirements and the prevailing vision in countries, especially where the SDG framework sets higher requirements than the country’s development capacity. Under the constraints of a higher target threshold, the challenge remains as to how to formulate a feasible blueprint (Lu et al., 2015). Moreover, restricted by the inadequate technology and resource utilization efficiency, improvements in SDGs, including economic growth and social needs, often come with high environmental costs that put planetary boundaries at stake, which in turn negatively affects the overall achievement of SDGs (Hua et al., 2020; O’Neill et al., 2018). This emphasizes that upgrading technology is key to accelerating SDG progress and reducing the gap between goals and actual capabilities.

### Turbulent geopolitical environment undermines the SDG process

Amid the pandemic and political conflicts, a peaceful external environment and functioning multilateral partnerships are critical to advancing the SDGs. Current geopolitical conflicts between countries are adding uncertainty to the SDG process (UN, 2020b). Armed conflict and regional instability have caused widespread cropland abandonment, further negatively affecting food security. It is estimated that cultivated croplands in war-ravaged South Sudan reduced by 16% from 2016 to 2018 (Olsen et al., 2021). Trade wars between major economies led to a surge in soybean cultivation in Brazil, which has led to deforestation, overuse of agricultural land, and thus threatening carbon sequestration (Aguiar et al., 2016; Macedo et al., 2012). Geopolitical conflict, more especially the war in Ukraine in 2022 (Osendarp et al., 2022), has further undermined international markets and threatened food and energy security, constraining international cooperation that would promote the SDGs (Mach et al., 2019). Implementing SDGs depends largely on financial support, especially for developing countries with large infrastructure needs and funding gaps. Under the prevailing turbulent geopolitical environment, aid funding to developing countries is likely to be further reduced, leaving them even less able to fulfill their financial needs (UN, 2020a). This highlights the importance of global coordination to extend sources of aid funds, cultivate social capital, and improve governance.

### Imbalances and trade-offs limit the effectiveness of SDG implementation

**Fig. 1:**
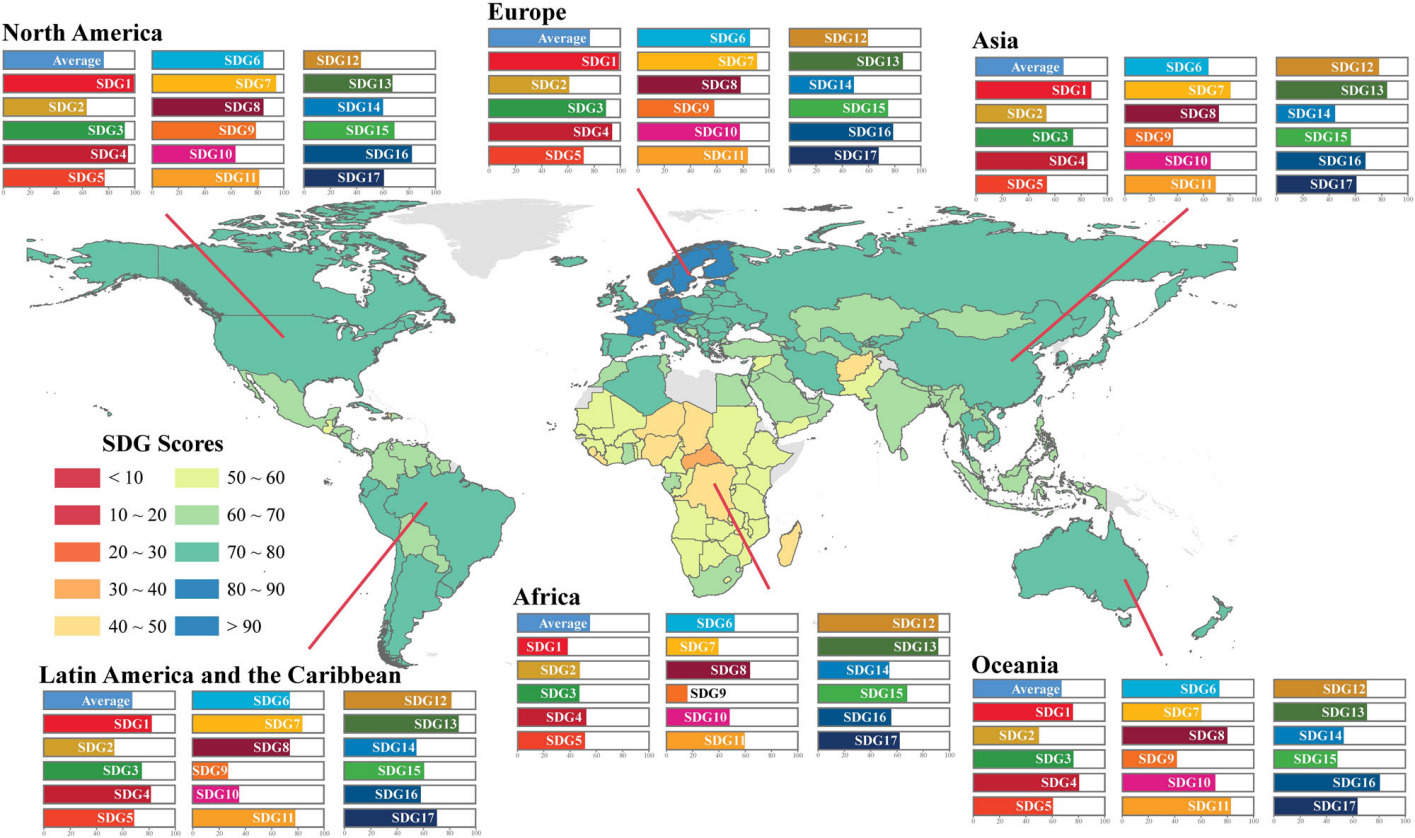
SDG performance scores in regions before the outbreak (2019). The color of the map reflects the global average score for each country on the 17 SDGs. Bar charts indicate the SDG index scores for different subregions, including Asia, Africa, North America, Europe, Latin America, the Caribbean, and Oceania. We collected data from the Sustainable Development Report 2019 (https://www.sdgindex.org/reports/sustainable-development-report-2019/).

Due to differences in the global resource base, status of the environment, and domestic and international political and economic circumstances, progress in advancing the SDGs is unbalanced and exhibits trade-offs globally (Figs. 1, S1, and S2b). Overall, in developing countries, the risk of climate change and deficiencies in the availability of even basic needs (e.g., clean water, energy availability) are key challenges (Balasubramanian, 2018; Cheng et al., 2021), while for developed countries, responsible consumption and production, coupled with ecosystem integrity are the main bottlenecks (Yang et al., 2020). Additionally, the Matthew effect is manifested in differential SDG achievement, as lower income-level nations evidently make slower progress in implementing the SDG agenda (UN, 2020b). Uneven SDG advancement, therefore, exacerbates development disparities between and within nations (Liu et al., 2021; Xu et al., 2020). On the other hand, the effectiveness of SDG actions and policies is affected by interactions between the goals and targets, and the extent to which synergies among them can be leveraged (Nilsson et al., 2018; Pradhan, 2019; Pradhan et al., 2017). Previous studies have highlighted negative interactions, for example between SDG10, SDG12, SDG13, and other goals that impede overall progress (Kroll et al., 2019; Pradhan et al., 2017). Such trade-offs appear to be even more pronounced in high-income countries. As SDG interactions evolve over time, synergies appear to be diminishing and trade-offs increasing, particularly in the case of interactions between SDG7 and SDG1, and SDG7 and SDG3 (Kroll et al., 2019). These perturbing findings mean that the current level of action towards implementing the SDGs is failing to leverage synergies and needs to be strengthened to overcome resistance induced by trade-offs.

### The effects of COVID-19 on SDG progress

#### COVID-19 leads to a decline in SDG performance

The COVID-19 pandemic has constrained progress in achieving the SDGs, as clearly illustrated by the marked decline in global SDG index scores in 2020 (Fig. S2a) and the increase in global poverty (SDG1) for the first time in decades, whereby an additional 119–124 million people fell back into extreme poverty (UN, 2020b). The impact of COVID-19 on population health and wellbeing, along with the disruption of medical services, may have reversed decades of SDG 3 progress (Ranjbari et al., 2021). In addition, more than 1.52 billion children and youth were out of school or university in 2020 under lockdowns, wiping out 20 years of education gains (SDG 4) (UN, 2020b). During the pandemic, the world faced the worst economic recession since the Great Depression (Ibn-Mohammed et al., 2021) and precipitated the loss of about 255 million full-time jobs (SDG 8) (UN, 2020a). The supply chain disruptions under lockdown conditions stalled the manufacturing industry and, GDP per capita was estimated to fall by 4.2% globally in 2020 (UN, 2020b). Meanwhile, the aviation industry suffered its steepest decline in history (Dube et al. 2021) as the number of air passengers fell by 60% from 4.5 billion in 2019 to 1.8 billion in 2020 (SDG 9) (UN, 2020b, 2021a).

#### COVID-19 amplifies unevenness in SDG progress

**Fig. 2:**
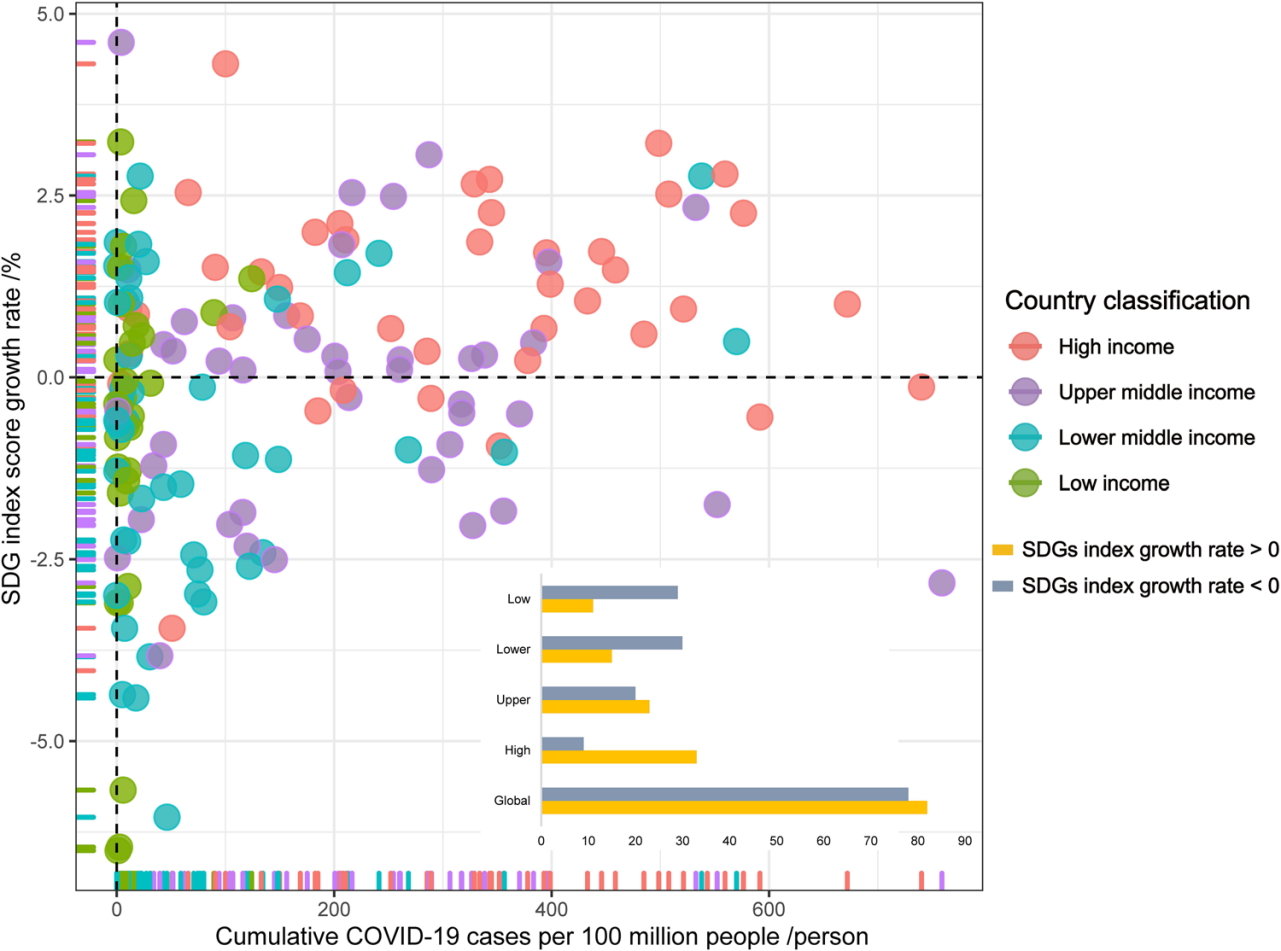
The relationship between the confirmed COVID-19 infection rate (cumulative cases per 100 million people in 2020) and SDG index score growth rate (2020 compared to 2019) globally. The bar chart shows the number of countries (divided into global, high-income, upper-middle-income, lower-middle-income, and low-income countries) achieving positive and negative SDG growth (2020 compared to 2019), respectively. We collected data from the Sustainable Development Report 2020 (https://www.sdgindex.org/reports/sustainable-development-report-2020/) and the WHO Coronavirus (COVID-19) Dashboard (https://covid19.who.int/).

COVID-19 has exacerbated differences among countries and communities in achieving the SDGs (Barbier and Burgess, 2020). Overall, the SDG index in almost half (49%) of the world’s countries has registered negative growth (i.e. SDG index score growth rate < 0, Fig. 2). Data show that SDG progress decreased significantly in most low and lower-middle-income countries, while it increased in high income countries (Fig. 2). Notwithstanding high COVID-19 confirmed rates in middle- and high-income countries, progress towards SDGs in 2020 has remained the same or, in some cases, actually improved (Fig. 2), suggesting that health systems, infrastructure, market and regulatory systems in such countries are more resilient to emergencies like COVID-19. Meanwhile, low-income countries lack the fiscal freedom to finance an adequate COVID-19 response and invest in recovery plans (Barbier and Burgess, 2020; UN, 2021b). It has been estimated that COVID-19 increased the average Gini coefficient (SDG 10) in emerging market and developing countries by 6% (UN, 2021a). Moreover, the risk of COVID-19 transmission has been shown to be much greater in densely populated urban areas (SDG11), which account for more than 90% of confirmed COVID-19 cases (UN, 2020a), more especially in developing countries where high density informal settlements are abundant. COVID-19 also had an uneven impact on different socio-economic groups, since the poor and more vulnerable, including women, children, the elderly, and informal workers, have been the hardest hit (Hawkins et al., 2020). Women comprise up to 70% of the global health workforce, making them more susceptible to infection (UN, 2021a), while increased care responsibilities and domestic violence during home isolation also threatened progress towards gender equality (SDG 5) (Chattu and Yaya, 2020). Older and homeless people were not only besieged by greater health risks but also likely to be less capable of supporting themselves in isolation (D’Cruz and Banerjee, 2020; UN, 2020a).

#### COVID-19 undermines connectivity and intensifies anti-globalization

The pandemic has delivered an unprecedented shock to the interconnected global system. Firstly, COVID-19 lockdowns directly limited inter-regional connectivity (such as the flow of human capital, and material and financial resources), which seriously affected the interconnected globalized economy and demand-supply chains (Dube et al.,^ 2021^; Galvani et al., 2020). COVID-19 has disrupted the global market, retarding progress towards economic growth (SDG 8) and production and consumption (SDG 12) in 80% and 70% of the world’s countries, respectively (Fig. S3). Secondly, the global lockdown has further disrupted social–ecological interactions. As the global recession loomed, local populations in developing countries with limited incomes were more likely to raid adjacent natural ecosystems, resulting in deforestation, poaching, overfishing, and the weakening of restoration initiatives (Roe et al., 2020). Indeed, the slump in ecotourism (SDG 9) during lockdown not only led to the loss of local income but also threatened ecological protection funds considered essential for wildlife and ecosystem conservation (SDG 15) (Naidoo and Fisher, 2020; Yin et al., 2021). Thirdly, in the absence of secure and stable international trade, many countries/regions were forced to depend more on domestic markets to maintain the demand for food, energy, and water (SDG 2, 6, 7), which in turn interrupts the food–energy–water nexus under conditions of limited ecological carrying capacity (Yin et al., 2022). Moreover, the pandemic disrupted global economic exchanges fueled regional inequality and disputes and exacerbated anti-globalization. Regional conflicts (such as wars, protests against structural inequality, and racism) intensified during COVID-19 (Cole and Dodds, 2021). Rising trade protectionism and anti-globalization have threatened both equality and peace (SDG 10, 16). Since multilateral and global partnerships are challenged by meager financial resources and regional conflicts, COVID-19 also impeded global partnerships, and, hence, SDG 17 progress has declined in 60.6% of the world’s countries (Fig. S3).

#### COVID-19 generates positive signals of change for SDGs

The pandemic not only posed challenges but also opportunities to promote sustainable transformation (Ibn-Mohammed et al., 2021; Pradhan and Mondal, 2021). Firstly, digital technology experienced a massive impetus and played a strongly supportive role, for example by improving healthcare responses, alleviating the impact of lockdowns, enabling business continuity through remote working, and supporting online education (Pan and Zhang, 2020). The digital economy and high-tech manufacturing fueled an economic recovery in late 2020, up 4% from the end of 2019 (SDG 9) (UN, 2021a). Secondly, while income constraints may have triggered the plundering of ecosystem resources, the drastic reduction in industrial emissions and economic activity reduced CO_2_ emissions and pollution, thereby improving environmental quality and ecosystem health in the short term (Le Quéré et al., 2020). COVID-19 was estimated to produce a 6% drop in greenhouse gas emissions for 2020 (SDG 13) (UN, 2021a). Limited human activity during the lockdown provided a chance for ecosystems and oceans to recuperate (SDG 14) (Diffenbaugh et al., 2020). Nevertheless, such improvements are likely to be only short-lived (climate action progress in 85.6% of countries declined throughout 2020, Fig. S3). Any such environmentally positive signals need to be prolonged by implementing sustainable development commitment once the pandemic ends and the global economy recovers.

### A systematic framework for promoting the achievement of SDGs in the post-pandemic era

To address the fragile foundations, uneven progress, and degraded cooperation context towards sustainable development and to help cope with the multiple challenges facing SDG progress before and amid COVID-19, we recommend that post-pandemic SDG actions adopt a systematic framework of “Classification–Coordination–Collaboration” (Fu et al., 2020).

#### Classification

Classification helps rationalize and identify the current development status, clarify the expected goals for sustainability, and select the appropriate development priorities and paths aligned with the national conditions.

1. *Elucidating the impact of the pandemic*: It is necessary for governments to adopt multiple solutions that deal with COVID-19’s adverse effects while maintaining positive momentum (Ranjbari et al., 2021). Containing the pandemic and avoiding further adverse social and economic impacts necessitates quick and effective interventions, not least the continuation of prevention, testing, and treatment measures but also through the effective roll-out of vaccines globally. Achieving herd immunity, in which a significant proportion of the population (70 percent) is vaccinated, remains an urgent requirement (Jean-Jacques and Bauchner, 2021). Improving health care systems, ensuring resource security, restoring livelihoods and production are also critical.
2. *Identifying the urgency of SDGs*: Some SDG targets are more urgent amid COVID-19 (e.g., medical and healthcare systems, food and water security) and, under the ongoing pandemic and economic recession, cash-strapped governments need to identify urgency and priorities among diverse SDG targets according to challenges and national development needs (Naidoo and Fisher, 2020). As noted above, identifying and harnessing the SDG synergies can enhance the effectiveness of SDG actions and policies. Based on the principles of cost–benefit and social–ecological analysis, governments, and experts need to determine win-win solutions (e.g., Nature-based Solutions) that minimize trade-offs, promote synergies, and address multidimensional development issues (Zhang et al., 2020), so as to meet their most urgent needs and to systematically promote the SDGs (Barbier and Burgess, 2020).
3. *Classifying countries/regions standards for SDGs*: COVID-19 has amplified deficiencies in the foundations of development, and inadequate resilience and recovery potential. It is difficult for less developed countries to achieve SDGs that are measured against a unified global standard, which therefore frustrates their efforts towards making progress. To implement targeted optimization measures for the SDGs, it would be helpful for the UN to classify different countries/regions, considering their differences in SDG baseline levels, resources, infrastructure, technology, and socio-economic status. More realistic and practicable SDG actions require common but differentiated targets for countries/regions at different levels of development. Countries within similar categories can establish national plans to take concerted action in terms of policy, finance, trade, infrastructure, and values.

#### Coordination

Coordination includes domestic and international support and inter and intra-departmental coordination for both short-term recovery and long-term development.

1. *Domestic and international coordination*: The global experience of COVID-19 suggests that stronger domestic and international coordination is needed for achieving SDGs. On one hand, countries need to establish their own management systems to ensure the security of basic resources (Wang et al., 2020), such as improving infrastructure and logistics systems and strengthening the domestic production and stock capacity. On the other hand, international organizations need to strengthen their coordination capacity to improve international dialog mechanisms, build platforms for bilateral and multilateral investment and trade cooperation, and coordinate market rules for basic resources, especially given the additional stresses brought about by the pandemic and associated impacts.
2. *Coordination between different administrative departments*: To enhance the synergistic effect among SDGs, it is vital that both intra- and inter-departmental coordination be reinforced. While the SDGs were originally presented and envisaged as separate goals, it is clear that they are strongly interdependent. Coordination between different target-related departments is key to avoiding policy overlap and conflict for SDGs. Facing financial and energy constraints after COVID-19, governments have the duty to seek solutions that minimize trade-offs, promote synergies, and address multidimensional development issues to systematically promote the SDGs.
3. *Coordination between short-term recovery and long-term development*: To set the goals back on the path to recovery, both short-term and long-term issues must be identified and addressed with targeted measures. In the short term, the pandemic has negatively impacted goals related to reducing poverty and securing food, water, and energy, which must be addressed urgently. However, in the longer term, improving infrastructure, promoting scientific and technological innovation, and optimizing the relationship between humans and nature are imperatives for the realization of the SDGs (Yang et al., 2020). Accordingly, in formulating socio-economic development policies for the post-pandemic era, attention of researchers and policymakers needs to be applied to both short-term recovery and long-term development.

#### Cooperation

Cooperation includes strengthening economic cooperation, promoting technological innovation, and jointly building a global culture of sustainable development.

1. *Economic cooperation to strengthen global economic partnership*: Different stakeholders urgently need to strengthen complementarity, adopt common but differentiated responsibilities and build a stable global economic environment. We recommend three actions for all relevant stakeholders as follows: (i) Provide targeted economic assistance to vulnerable communities/regions in a way that allocates aid funds scientifically, thus ensuring their more efficient and equitable use. (ii) Deepen international cooperation on production capacity by implementing actions that optimize production modes, improve production efficiency and economic benefits, increase employment, and avoid resource waste and environmental damage. (iii) Jointly build a platform for financial cooperation by expanding access to financing to strengthen the resilience of global markets.
2. *Technological cooperation to promote scientific and technical innovation*: If economies ravaged by the pandemic are to get back on track, there is an urgent need for researchers, corporations, and the technology industry to strengthen the exchange of scientific and technical innovation in the following areas: (i) Building platforms for collaboration that establish channels for joint innovation and technology transfer between countries/institutions while protecting intellectual and rights. (ii) Ensuring that funding for innovation and collaboration flows into key scientific and technical fields, thereby supporting original scientific achievements and guiding market funds towards them. (iii) Cultivating international talents to stimulate innovation and unleash the greater potential of scientific and technological innovation through collaboration.
3. Cooperation between cultures to shape sustainable development values. Repairing cultural isolation caused by COVID-19 and the lockdown is even more critical than ever if the world is to recover from the pandemic. On the basis of respecting cultural diversity, cultural institutions, and organizations need to take action to form and nurture a culture of global sustainability and values. Effective actions towards this include: (i) deepening and broadening access to education for sustainable development to enhance public understanding, recognition, and willingness to participate in SDGs, respect differences and diversity, and foster the values of harmony with nature. (ii) Improving the development and maintenance of a cultural infrastructure that reduces cultural imbalances that aggravate gender and racial discrimination and foment violent conflicts.

## Concluding remarks

COVID-19 has delivered a grave shock to SDG progress. Under global lockdowns and economic recession, political disputes and armed conflicts constrain international coordination and cooperation for sustainable development. In addition, multiple pressures continue to threaten the achievement of SDGs, and their implementation is not simply a case of stepping out of the shadow of the pandemic, since ongoing population growth puts increased pressure on limited natural resources, climate change impacts social security and intensifies natural disasters, and ecosystem degradation and pollution shake the very foundations of sustainable development. We, therefore, urge the UN’s High-level Political Forum, national governments, researchers, and stakeholders to consider more seriously how to recover and transform SDG actions after the pandemic. In the post-pandemic era, it is urgent that more systematic solutions be found and adopted. Such solutions can be enhanced by reclassifying the SDG status and vision, coordinating multiple resources and policies, and promoting and consolidating economic, technological, cultural, and political collaboration. Last but not least, with just 8 years to 2030, we have to plan ahead regarding where and how SDGs should evolve in 2030 and after 2030. For the phase after 2030, the SDG vision needs to be oriented towards 2045, a year which, incidentally, marks the UN’s 100th anniversary. Ideally, the pandemic and global recession will in the future be considered a watershed, and Agenda 2030 is a milestone guiding the world towards Agenda 2045 and a truly sustainable future.

## Supplementary information

Supplementary Materials: Achieving the Sustainable Development Goals in the post-pandemic era

## Data Availability

The datasets generated and analyzed in this study are available from the corresponding author on reasonable request.
