# Supplementary material for: Achieving the Sustainable Development Goals in the post-pandemic era
Source: Humanit Soc Sci Commun. 2022 Aug 6;9(1):258. doi: 10.1057/s41599-022-01283-5 (PMC9362700; doi:10.1057/s41599-022-01283-5)
Supplement: Supplementary file 1 — Supplementary Materials: Achieving the Sustainable Development Goals in the post-pandemic era [file 41599_2022_1283_MOESM1_ESM.pdf]

Supplementary Materials

Achieving the SDGs in the post-pandemic era

Wenwu Zhao[1,2,], Caichun Yin[1,2], Ting Hua[1,2], Michael E Meadows[4,5,6], Yan Li[1,2], Yanxu Liu[1,2], Francesco Cherubini[7], Paulo Pereira[8], Bojie Fu[1,2,3*]

* Corresponding author: bfu@rcees.ac.cn

[1] State Key Laboratory of Earth Surface Processes and Resource Ecology, Faculty of Geographical Science, Beijing Normal University, Beijing, China

[2] Institute of Land Surface System and Sustainable Development, Faculty of Geographical Science, Beijing Normal University, Beijing, China

[3] State Key Laboratory of Urban and Regional Ecology, Research Center for Eco-Environmental Science, Chinese Academy of Sciences, Beijing, China

[4] Department of Environmental & Geographical Science, University of Cape Town, Rondebosch, South Africa

[5] School of Geographic and Ocean Sciences, Nanjing University, Nanjing, China

[6] College of Environmental Sciences, Zhejiang Normal University, Jinhua, China

[7] Industrial Ecology Program, Department of Energy and Process Engineering, Norwegian University of Science and Technology, Trondheim, Norway

[8] Environmental Management Center, Mykolas Romeris University, Vilnius, Lithuania

This file includes:

Figure S1. Global SDGs' performance before the outbreak

Figure S2. SDG index score

Figure S3. The relationships between the confirmed COVID-19 rate and 17 SDGs' index score growth rate globally

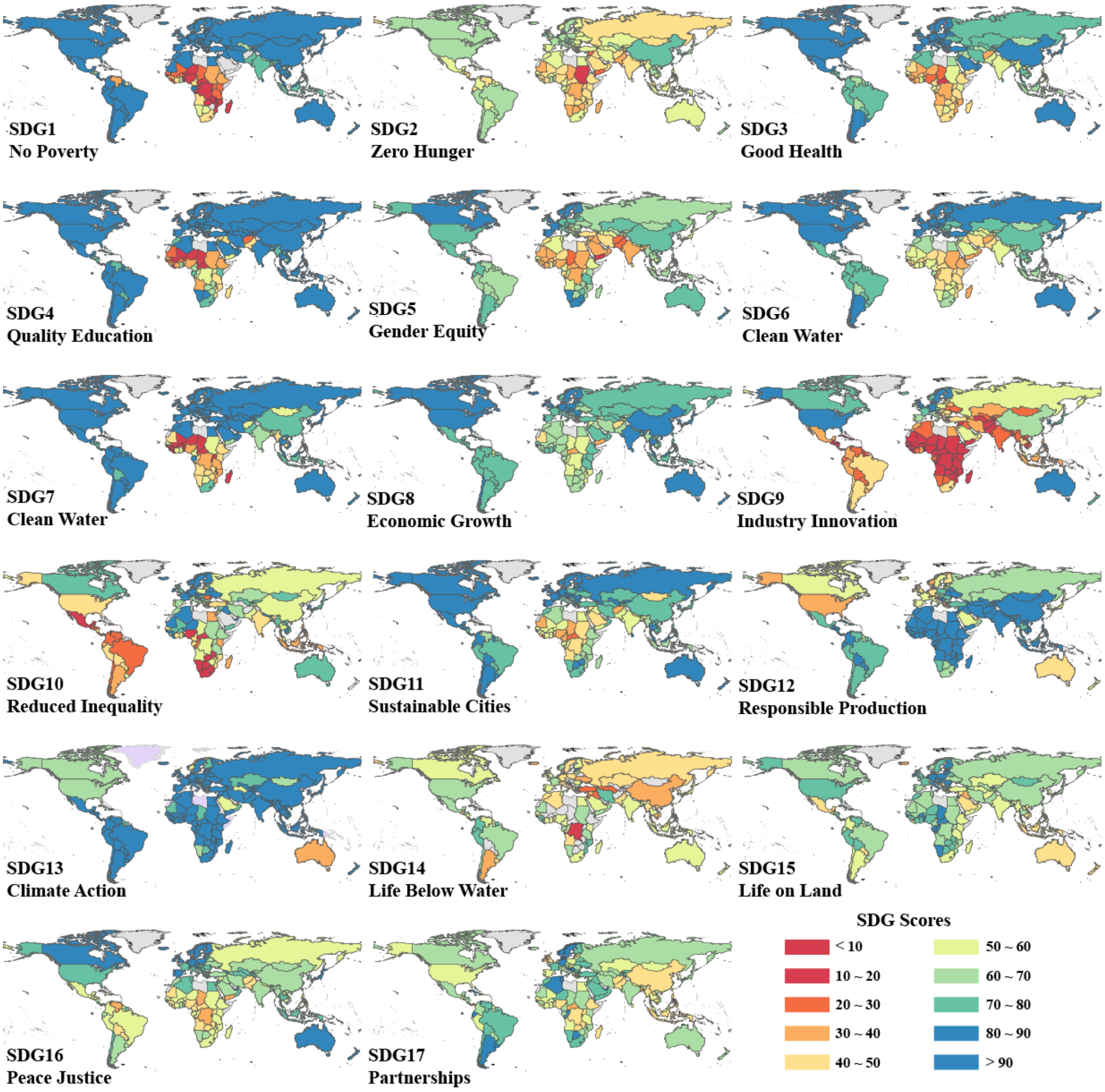

Figure S1. Global SDGs' performance before the outbreak. We used SDG scores in 2019 to characterize SDGs' performance before the outbreak. Data were derived from Sustainable Development Report 2019 (https://www.sdgindex.org/reports/sustainable-development-report-2019/). Colors reflect scores for the different SDGs for each country according to the legend.

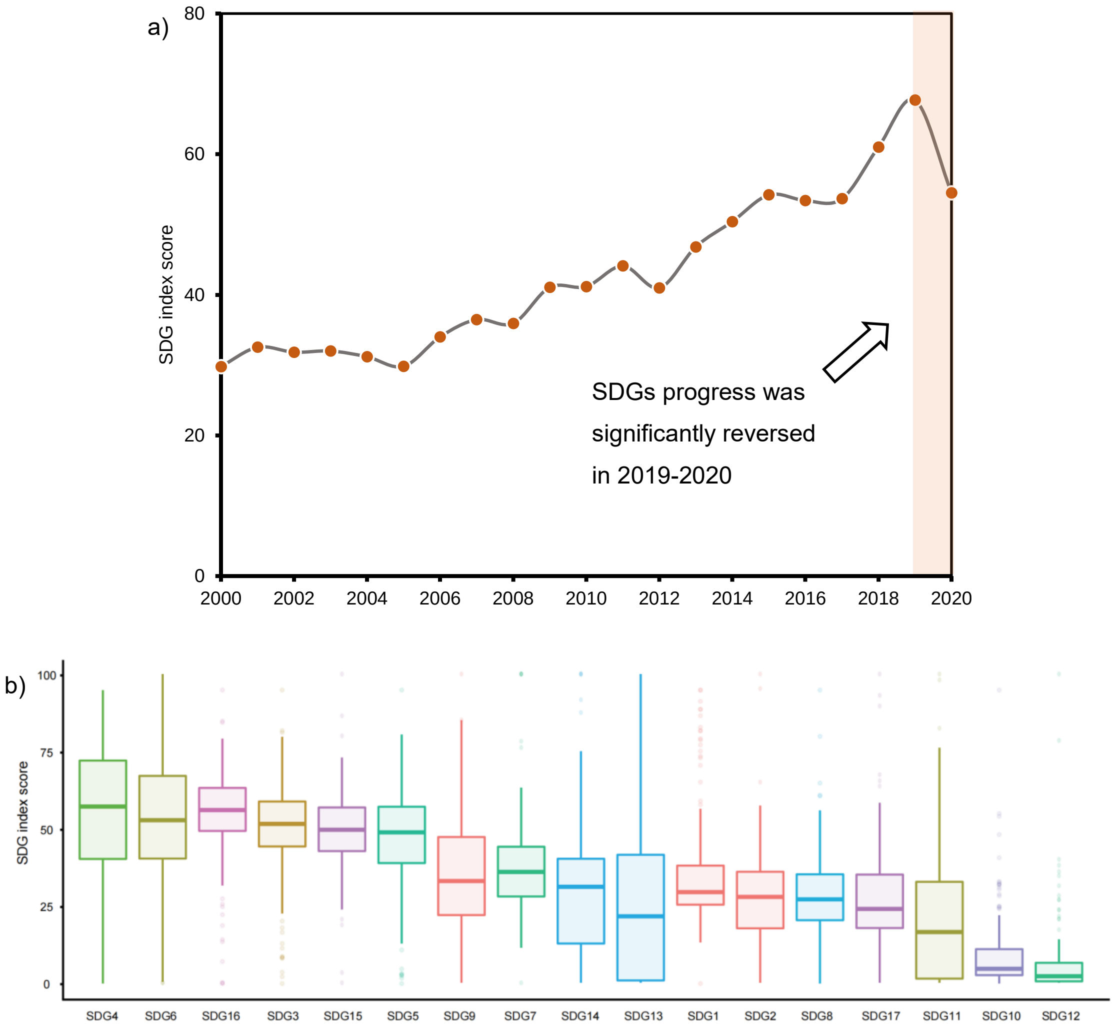

Figure S2. SDG index score. a) Global SDG index score from 2000 to 2020. b) Average SDG index score of 17 SDGs. The boxplot shows the minimum value, first quartile, median, third quartile, maximum value and outliers of SDG index score. Relevant data were obtained from Sustainable Development Report 2020 (https://www.sdgindex.org/reports/sustainable-development-report-2020/).

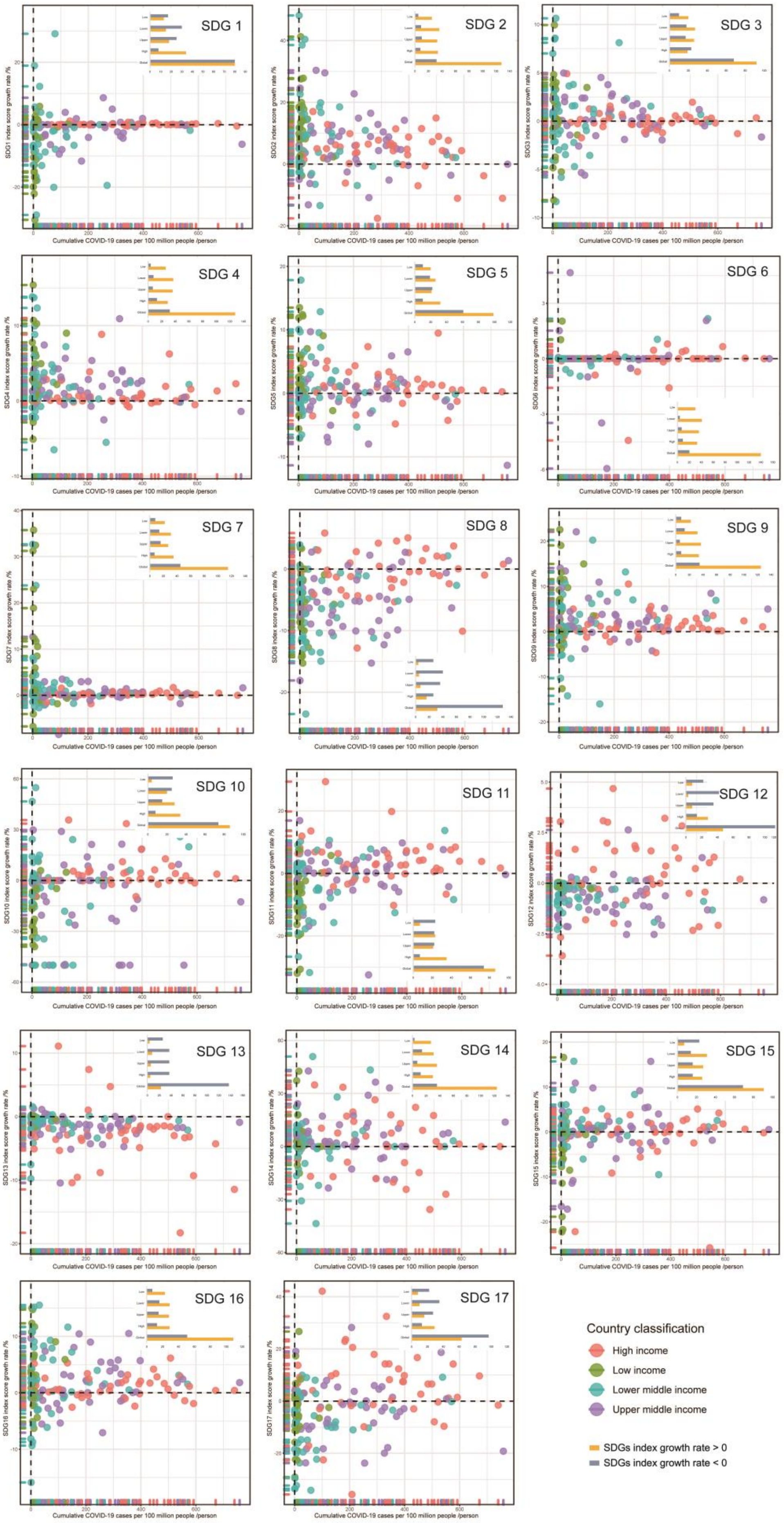
SDG 1
SDG 2
SDG 3
SDG 4
SDG 5
SDG 6
SDG 7
SDG 8
SDG 9
SDG 10
SDG 11
SDG 12
SDG 13
SDG 14
SDG 15
SDG 16
SDG 17
Cumulative COVID-19 cases per 100 million people /person
Country classification
High income
Low income
Lower middle income
Upper middle income
SDGs index growth rate > 0
SDGs index growth rate < 0

Figure S3. The relationships between the confirmed COVID-19 infection rate (cumulative cases per 100 million people in 2020) and SDG index score growth rate (2020 compared to 2019) globally. The bar chart shows the number of countries (divided into global, high-income, upper middle-income, lower middle-income and low-income countries) achieving positive and negative SDG growth (2020 compared to 2019) respectively. Relevant data were obtained from the Sustainable Development Report 2020 (https://www.sdgindex.org/reports/sustainable-development-report-2020/) and the WHO Coronavirus (COVID-19) Dashboard (https://covid19.who.int/).
